# Emergency Department Screening for Unhealthy Alcohol and Drug Use with a Brief Tablet-Based Questionnaire

**DOI:** 10.1155/2020/8275386

**Published:** 2020-07-17

**Authors:** Joshua W. Elder, Evan F. Wu, James A. Chenoweth, James F. Holmes, Aman K. Parikh, Aimee K. Moulin, Tommie G. Trevino, John R. Richards

**Affiliations:** Department of Emergency Medicine, University of California Davis Medical Center, Sacramento, CA, USA

## Abstract

**Background:**

Screening for unhealthy alcohol and drug use in the emergency department (ED) can be challenging due to crowding, lack of privacy, and overburdened staff. The objectives of this study were to determine the feasibility and utility of a brief tablet-based screening method in the ED and if patients would consider a face-to-face meeting with a certified alcohol and drug counselor (CADC) for more in-depth screening, brief intervention, and referral to treatment (SBIRT) helpful via this interface.

**Methods:**

A tablet-based questionnaire was offered to 500 patients. Inclusion criteria were age ≥18, Emergency Severity Index 2–5, and English comprehension. Subjects were excluded if they had evidence of acute intoxication and/or received sedating medication.

**Results:**

A total of 283 (57%) subjects were enrolled over a 4-week period, which represented an increase of 183% over the monthly average of patients referred for SBIRT by the CADC prior to the study. There were 131 (46%) who screened positive for unhealthy alcohol and drug use, with 51 (39%) and 37 (28%) who screened positive for solely unhealthy alcohol use and drug use/drug use disorders, respectively. There were 43 (33%) who screened positive for combined unhealthy alcohol and drug use. Despite willingness to participate in the tablet-based questionnaire, only 20 (15%) with a positive screen indicated via the tablet that a face-to-face meeting with the CADC for further SBIRT would be helpful.

**Conclusion:**

Brief tablet-based screening for unhealthy alcohol and drug use in the ED was an effective method to increase the number of adult patients identified than solely by their treating clinicians. However, only a minority of subjects screening positive using this interface believed a face-to-face meeting with the CADC for further SBIRT would be helpful.

## 1. Introduction

Unhealthy alcohol use, defined as the presence of an alcohol use disorder, as determined by a standardized diagnostic interview, or risky consumption, as determined using a validated 30-day calendar method, and drug use and drug use disorders are common causes of emergency department (ED) visits [1–4]. This patient subgroup not only is overrepresented in the ED but also utilizes substantial amounts of ED services [1–6]. In 2010, the United States Centers for Disease Control and Prevention (CDC) estimated annual medical costs of alcohol use disorder alone to be $249 billion [7]. Both the National Institute of Health (NIH) and the Substance Abuse and Mental Health Services Administration (SAMHSA) report clear trends of rising costs of alcohol use disorder [8, 9]. Similarly, annual costs from the use of illicit drugs and misuse of prescription drugs have also risen [8, 10–12]. Another serious consequence is that deaths from drug overdose continue to increase in the United States, with over 702,000 fatalities from 1999 to 2017 [13]. In 2017 alone, more than 70,000 people died from drug overdoses, of which 68% involved opioids. Drug overdose is now the leading cause of injury-related death in the United States [13].

Screening, Brief Intervention, and Referral to Treatment (SBIRT) is a public health framework method developed to identify alcohol and drug users, motivate, and refer them to rehabilitation and recovery services [14]. This evidence-based approach has been shown to be efficacious in ED settings [15–19]. Most initial screening is performed by clinicians caring for ED patients, who have variable training and interest in the longitudinal care of patients with alcohol and drug use disorders. However, several EDs have implemented certified alcohol and drug counselors (CADC) to evaluate patients using SBIRT, alcohol use disorder identification test (AUDIT), and drug abuse screening test (DAST) [20–22]. Based on patient responses, the CADC can conduct brief interventions, provide motivation for rehabilitation, and arrange referral to treatment from the ED. Unfortunately, the majority of EDs in the United States lack dedicated CADCs or do not have 24-hour coverage of such services. As a result, initial identification of patients in the ED can be challenging secondary to ED crowding, lack of patient privacy, and provider time constraints.

At the study site ED, patients are routinely screened for domestic abuse and suicide at initial intake by a registered nurse, but screening for unhealthy alcohol and drug use was not routinely performed. Six months prior to this study, there was no SBIRT screening and/or CADC presence in the ED. Instead, ED patients with unhealthy alcohol and drug use were discharged by their treating clinicians with paper information leaflets (“street sheets”) on how to arrange their own outpatient follow-up, with contact information for various recovery and rehabilitation programs in the area. Although not specifically measured, the success of this type of screening and referral was believed by ED clinicians and nurses to be limited.

Due to the entropic nature of the ED, clinician screening and identification of patients with unhealthy alcohol and drug use and their referral to outpatient rehabilitation services at discharge are often inefficient and inconsistent and may miss a substantial proportion of patients. Given the challenges unique to a busy ED, a brief tablet-based screening questionnaire may better optimize identification of these patients than current provider-directed processes. The objectives of this study were to determine the feasibility and utility of a brief tablet-based screening questionnaire in the ED and to determine patient attitudes towards subsequent face-to-face meeting with the CADC for SBIRT via this method.

## 2. Methods

This was a prospective, cross-sectional study of patients at a university-affiliated, urban Level I trauma and tertiary care medical center serving northern and central California. The study site ED has a surrounding metropolitan area of two million residents, an annual census of approximately 85,000 patients, and an associated residency training program in Emergency Medicine. The study site also serves as the *de facto* safety net for underserved, undocumented, and uninsured patients, especially those requiring acute psychiatric care. This ED also contractually accommodates victims and perpetrators brought by law enforcement. After the advent of the dedicated in house CADC six months prior to this study, an average of 100 ED patients per month were identified using varied and unvalidated subjective and objective methods by their treating clinician as possibly having unhealthy alcohol and drug use. Many of these patients were intoxicated and incapable of accurate screening at this point in their ED care, or they arrived after business hours or on the weekend. They were then placed on a shared electronic medical record (EMR) list to undergo face-to-face SBIRT by the CADC when they were no longer intoxicated. If the patient acquiesced or had not left the ED after being placed on the shared EMR list, the CADC then performed SBIRT based on the list on weekdays from 8 AM to 5 PM.

The study was approved by the site's Institutional Review Board as an exemption with the caveat that any potential patient identifiers must not be included in the questionnaire. To comply with this constraint, the demographic data from the questionnaire was limited to gender. Subject inclusion criteria were as follows: (1) age ≥ 18 years, (2) Emergency Severity Index (ESI) triage acuity category 2–5 (with category 1 representing an immediate life-threatening illness or injury), and (3) English comprehension. Patients were excluded if there was any evidence of acute intoxication and/or having received sedating medication while in the ED. Patients completed the questionnaire once and were excluded if they had previously completed it.

**Figure 1:**
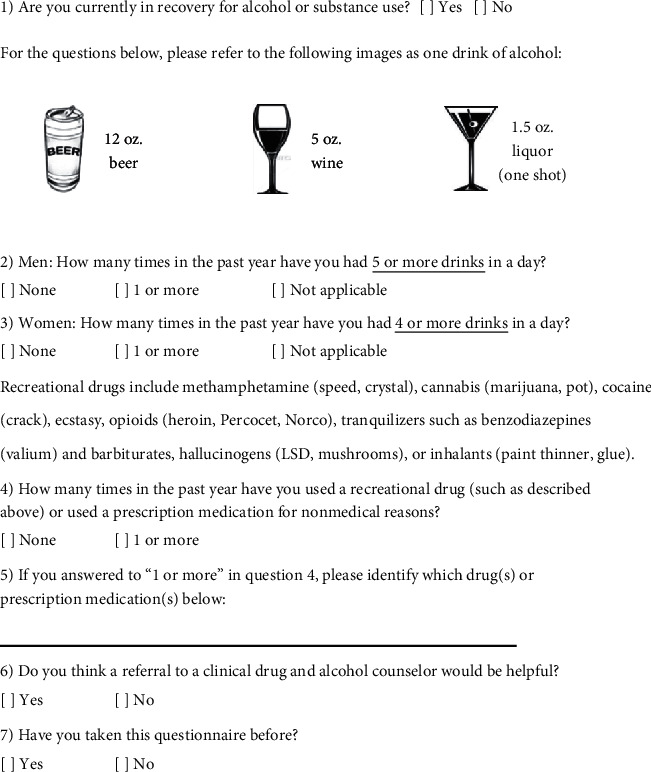
Tablet-based drug and alcohol screening questionnaire.

During the continuous 4-week study period of November 19 to December 16, 2018, a convenience sample of ED patients were instead approached by members of the Emergency Medicine Research Associate Program (EMRAP), a group of undergraduate students trained in the identification and enrollment of patients in ED-based clinical research studies. The EMRAP associates identified and enrolled subjects during specific hours of the day (5 AM to 11 PM) seven days a week. The patient questionnaires (Figure 1) were accessed through a tablet-based Research Electronic Data Capture (REDCap, Vanderbilt University, Nashville, Tennessee, USA) online questionnaire. REDCap is a Health Insurance Portability and Accountability Act (HIPAA) compliant, secure web application that allows users to create a research database from web-based forms linking data with statistical software and other analytical tools. This study utilized the Amazon Fire 7 tablet (Quanta Computer, Taoyuan City, Taiwan). Questionnaire responses and completion time measured in minutes were recorded and entered automatically to the REDCap database.

The brief screen was derived from two previously published studies by Smith and colleagues assessing the utility of single question screening tests for unhealthy alcohol and drug use [23, 24]. A follow-up question clarifying the identity of illicit drug(s) was posed if subjects affirmed drug use on the screening questionnaire. Subjects were next queried about active recovery for unhealthy alcohol and drug use, as these patients may not require the full spectrum of CADC resources. Subjects were then asked if a face-to-face meeting with the CADC for SBIRT would be helpful. Finally, subjects were queried if they had taken the questionnaire before. Images and question format were modelled after the Oregon SBIRT [25]. Patients who initially consented to the tablet-based questionnaire could stop at any point if they desired. Differences in proportion between subgroups were analyzed using chi-square (*X*^2^) test with Yates correction (MedCalc version 19.1, Ostend, Belgium). Statistical significance was assumed at a level of *P* ≤ 0.05.

## 3. Results

A total of 283 (57%) of 500 ED patients who fulfilled the inclusion criteria and were approached by EMRAP associates completed the tablet-based questionnaire. This represented an increase of 183% over the previous monthly average of patients identified by treating clinicians and placed on the shared EMR list to undergo SBIRT by the CADC (*n* = 100). Of the 283 study subjects, 148 (52%) were male. Nine subjects (2%) started but did not complete the questionnaire. There was no significant difference in gender proportions between those who completed the questionnaire and those who declined (52% versus 60% male, *X*^2^ = 2.5, *P*=0.1). The time to complete the questionnaire was less than one minute for all subjects. A positive screen for unhealthy alcohol and drug use was identified in 131 (46%) subjects of whom 51 (39%) screened positive for only unhealthy alcohol use and 37 (28%) for drug use/drug use disorders. There were 43 (33%) subjects who screened positive for combined unhealthy alcohol and drug use. When patients who screened positive were combined with those patients identified by their treating clinicians and placed on the shared EMR list, this represented an increase of 131% of total patients who could potentially benefit from SBIRT provided by the CADC.

There were significant gender differences between subjects with positive and negative screens for unhealthy alcohol and drug use, with a higher proportion of males screening positive compared to negative (65% versus 41%, *X*^2^ = 15.5, *P* < 0.0001). Within each positive screen subgroup (alcohol, drugs, alcohol, and drugs), the proportion of males was higher than females: alcohol: *n* = 29 (57%) male; drugs: *n* = 24 (65%) male; alcohol and drugs: *n* = 32 (74%) male. However, these differences did not reach statistical significance between the three positive screen subgroups (*X*^2^ = 3.2, *P*=0.2).

Of the subjects who screened positive for drug use disorders (*n* = 80), commonly reported illicit drugs included marijuana (*n* = 57, 71%) and methamphetamine (*n* = 8, 10%). Co-use of both marijuana and methamphetamine was indicated by four subjects (5%). The use of three or more illicit drugs was noted by three subjects (4%). Other reported illicit drugs included cocaine (*n* = 3) and heroin (*n* = 2). Alprazolam, methylenedioxymethamphetamine (ecstasy), gamma-hydroxybutyrate (GHB), morphine, hydromorphone, hydrocodone, and methadone were reported singly.

Despite willingness to participate in the tablet-based questionnaire lacking human interaction, only 20 (15%) with a positive screen for unhealthy alcohol and drug use indicated that a face-to-face meeting with the CADC for SBIRT would be helpful to them. Between positive screen subgroups, the proportion of subjects who indicated that meeting with the CADC would be helpful was the highest in the combined unhealthy alcohol and drug use cohort (21%) compared to unhealthy alcohol use only (12%) and drug use/drug use disorder only (14%) cohorts; however, this was not statistically significant (*X*^2^ = 1.6, *P*=0.4). Fourteen subjects (11%) in the positive screen group indicated they were in a recovery program at the time of the tablet-based questionnaire, whereas two (1%) in the negative screen indicated the same.

## 4. Discussion

Screening patients with unhealthy alcohol and drug use in the ED is important to identify those at risk for withdrawal syndromes, minimize potential for deleterious drug interactions with anesthetics and prescribed psychotropic medications, decrease hospital length of stay, and arrange potential solutions for cessation. The current study evaluated the feasibility and utility of universal screening of ED patients for unhealthy alcohol and drug use with a brief tablet-based screening approach. The results demonstrate that patients are willing to participate in tablet-based screening, which involves no human interaction and can be successfully completed in a timely fashion. Though data regarding tablet-assisted screening for unhealthy alcohol and drug use is limited, other studies have demonstrated both feasibility and increased rates of detection [26–30]. This tool could effectively triage patients for further evaluation, which may include a SBIRT, AUDIT, or DAST. However, authors of a recently published scoping review of non-face-to-face SBIRT modalities, such as tablets, smartphones, and computers, reported mixed success with the intervention and referral components of SBIRT [31]. We believe our study contributes to the existing literature by demonstrating that despite the feasibility of rapid tablet-based screening for harmful alcohol and drug use to greatly increase the number of patients screened by solely their treating clinicians, willingness to pursue further face-to-face SBIRT with a CADC and perhaps downstream treatment after discharge may be limited via the tablet interface.

As mentioned earlier, patients at the study site are identified by their treating clinicians as potentially having unhealthy alcohol and drug use and placed on a shared EMR list to undergo SBIRT by the ED-based CADC. However, studies have shown inaccuracies and lower detection rates with provider-based methods of identification [32–36]. The brief tablet-based questionnaire enabled identification of unhealthy alcohol and drug use in 46% of patients. This method greatly increased the number of patients identified by their treating clinicians, which was previously measured as an average of roughly three patients per day. Another primary measure of feasibility was time spent to administer and complete the tablet-based screening questionnaire. All questionnaires required less than one minute to complete by the patient, and no additional clinician time was required to administer the questionnaire and input the responses into a database.

Tablet computers, such as the Amazon Fire and Apple iPad, appeared in 2010 and their use became widespread in the following years. Tablet computers are especially suited for patient use in the ED from portability, lack of an attached keyboard, and ability to disinfect the touchscreen after use. Specific tablet-based (not laptop or desktop computer) screening for harmful alcohol and drug use in the ED has been previously described, and these studies have some differences and similarities to our findings. A 2012 study of tablet Computerized Alcohol Screening and brief Intervention (CASI) of trauma patients using AUDIT was one of the first to describe the use of tablets in the ED to determine predictive factors for “hazardous drinking behavior” [37]. However, no mention was made of time to completion, proportion of patients who refused, or details of the tablet-based brief intervention. In another 2012 publication, this research group also described the use of CASI in the same trauma population to assess “readiness to change” [38]. Patients screening positive underwent a computer-guided brief negotiated interview that included personalized feedback, readiness to change, reasons for cutting down, goal setting, and a printed personal alcohol reduction plan. Half of patients screened indicated they were “ready to change” after completing the CASI, with some gender and ethnic differences, and this was much higher than the proportion of subjects in our study indicating that face-to-face SBIRT with the CADC would be helpful. In their second study, there was no mention of the proportion of patients who refused CASI.

The following year Murphy and colleagues reported using tablet-based screening using AUDIT to identify “at-risk alcohol users” in the ED, who were discharged with printed National Institute on Alcohol Abuse and Alcoholism (NIAAA) educational materials and a nationwide informational phone number for referral to treatment [27]. Thirty-two percent of patients in the sample screened positive for “at-risk drinking,” and 28% reported some intention to consult a healthcare professional about their alcohol use as a result of their screening results, which was similar to the proportion of subjects in our study who believed a face-to-face meeting with the CADC would be helpful. Another study the same year by Lotfipour et al. compared a triage nurse-administered medical screening examination to CASI and reported the tablet method was superior in identifying “at-risk alcohol drinkers” [26]. In our study, we did not compare provider-based identification with tablet-based screening, but we did find tablet-based screening greatly increased the number of patients identified as having potential benefit from further SBIRT by our CADC. In 2015 Cunningham and associates compared a 15-minute brief intervention using a tablet with headphones versus brief intervention with a therapist and found both to be nearly identical in reducing alcohol consumption index and consequences in underage drinkers [39]. Unlike our study, the authors first screened their subjects with AUDIT from the EMR and the tablet portion of the study involved brief intervention with financial compensation. Weiner et al. showed that tablet screening in the ED for “opioid abuse” using a revised Screener and Opioid Assessment for Patients with Pain (SOAPP) with 24 questions could be accomplished in less than 5 minutes [31]. Their study did not include follow-up with a CADC or other interventions or referrals after discharge from the ED.

More recently, Haskins and associates compared ED patients who completed a tablet-based SBIRT, the Health Evaluation and Referral Assistant (HERA), to minimal-treatment controls and found HERA promoted contact with an alcohol treatment provider and initiation of treatment via a faxed referral but did not reduce risky alcohol use behavior [40]. Similar to our study, a significant proportion of patients (33%) declined to participate in the study. Strzsak et al. reported the use of a tablet-based Alcohol, Smoking and Substance Involvement Screening Test (ASSIST) to determine the prevalence of “risky substance use” in the ED [41]. Similar to our study findings, 51% screened positive for risky substance use, and the mean time to completion was 5 minutes.

Patients who have unhealthy alcohol and drug use have variable attitudes towards intervention, cessation, and rehabilitation, which may change during their ED stay. Determining patients' viewpoints regarding downstream referral to rehabilitation and recovery services is an important step in their ED care. In this study, less than one out of every five subjects with a positive screen for unhealthy alcohol and drug use answered that a face-to-face meeting with a CADC would be helpful to them. Unfortunately, there was no real-time feedback to clarify or explain what was meant by this question for patients considering their answer while using the tablet. This invisible barrier may reflect a limitation of the tablet-based interaction, in that human interaction and feedback are missing. This lack of feedback may result in less willingness to seek in-person CADC assistance to arrange recovery and rehabilitation follow-up after discharge from the ED. Another potential explanation is that unhealthy alcohol and drug use is stigmatized, and patients may feel their overall medical care will be affected by also acquiescing to counseling and referral during their ED stay. As such, further investigation is needed to determine why most patients did not indicate a face-to-face meeting with the CADC would be helpful via the tablet interface.

## 5. Limitations

This study has several limitations. The number and availability of EMRAP coworkers were limited during the November and December holidays, which may have resulted in fewer available screeners and subjects enrolled than if the study had taken place during a different month with fewer holidays. Given the use of a convenience sample of only English-speaking subjects, selection bias may also play a role. Demographic data was limited, as the study protocol was approved as an exemption by our facility's Institutional Review Board due to patient confidentiality concerns. The study used self-reporting that was reliant upon participant responses, and thus recall bias is a concern. The wording of the question, “Do you think a referral to a clinical drug and alcohol counselor would be helpful?” may have been confusing to study subjects, and there was no way to clarify or explain via the tablet what a positive response would imply or entail later on.

## 6. Conclusion

The use of a tablet-based brief screening questionnaire is feasible and significantly increases the number of patients with unhealthy alcohol and drug use identified when compared to their treating clinicians. This method of screening should also improve ED flow and efficiency of patient care and lessen the burden on ED staff. In general, ED-based screening programs have the potential for increased referral rates for rehabilitation and recovery, which could also lead to less ED utilization and more reliance on outpatient services. Despite agreeing to participate in the tablet-based questionnaire, most patients who screened positive for unhealthy alcohol and drug use did not indicate a face-to-face meeting with a trained alcohol and drug counselor would help them. The next step is to determine how this technology can best be implemented to facilitate the “brief intervention” and “referral to treatment” aspects of SBIRT.

## Data Availability

Data were not made available since they contain potentially identifiable patient information.
